# Insecticide Resistance and Management Strategies in Urban Ecosystems

**DOI:** 10.3390/insects7010002

**Published:** 2016-01-06

**Authors:** Fang Zhu, Laura Lavine, Sally O’Neal, Mark Lavine, Carrie Foss, Douglas Walsh

**Affiliations:** 1 Irrigated Agriculture Research and Extension Center, Washington State University, Prosser, WA 99350, USA; soneal@tricity.wsu.edu (S.O.N.); dwalsh@wsu.edu (D.W.); 2 Department of Entomology, Washington State University, Pullman, WA 99164, USA; lavine@wsu.edu (L.L.); mark.lavine@wsu.edu (M.L.); 3 Puyallup Research and Extension Center, Washington State University, Puyallup, WA 98371, USA; cfoss@wsu.edu

**Keywords:** IPM, IRM, insecticide resistance, mechanism, molecular marker, genetically modified insect, RNAi-based insecticide, biopesticide

## Abstract

The increased urbanization of a growing global population makes imperative the development of sustainable integrated pest management (IPM) strategies for urban pest control. This emphasizes pests that are closely associated with the health and wellbeing of humans and domesticated animals. Concurrently there are regulatory requirements enforced to minimize inadvertent exposures to insecticides in the urban environment. Development of insecticide resistance management (IRM) strategies in urban ecosystems involves understanding the status and mechanisms of insecticide resistance and reducing insecticide selection pressure by combining multiple chemical and non-chemical approaches. In this review, we will focus on the commonly used insecticides and molecular and physiological mechanisms underlying insecticide resistance in six major urban insect pests: house fly, German cockroach, mosquitoes, red flour beetle, bed bugs and head louse. We will also discuss several strategies that may prove promising for future urban IPM programs.

## 1. Introduction

Entomologists face a diverse set of challenges to help protect humans and domesticated animals from urban insect pests. Continuing human population growth has been concurrent with increased urbanization. Today, more than 50% of the world’s population lives in cities, a proportion that will reach 70% by 2050 [1]. It is estimated that 6.3 billion people will live in urban areas by 2050. Entomologists will be tasked with developing sustainable practices to effectively control the urban insect pests that are closely associated with the health and quality of life for humans and domesticated animals.

Integrated pest management (IPM) was initially developed in the 1950s to promote a concerted use of chemical and biological approaches for pest control [2]. The concept of IPM was subsequently expanded to include the integration of biological, cultural and chemical tactics in a compatible manner to achieve favorable economic and environmental consequences. IPM aims to minimize the input of pesticides and reduce harmful effects of pesticides on non-target organisms and the environment. Today, IPM has become a fundamental strategy of sustainable agricultural arthropod pest management in developed and developing countries [3]. Development of a theory and practice of IPM in urban ecosystems that is parallel to IPM in agroecosystems is a pressing need among contemporary urban entomologists, pest control companies and stakeholders. The goal of agricultural IPM is to maintain the abundance of pests below an established economic injury level. Therefore, agriculture IPM is quantitative, objective and based on measureable metrics. In contrast, urban IPM is largely qualitative and subjective because it is based on many factors such as customer preconceptions regarding pest control and pesticide use. Additionally, the socioeconomic conditions of residents greatly contribute to the success or failure of urban IPM [4].

Insecticides are an essential part of an IPM program. In urban ecosystems, as in many agricultural systems and situations, insecticides are typically convenient, fast acting and inexpensive. Insecticides can be applied by pest management professionals or by household residents. Most residents of structures in urban settings are reluctant to cohabitate with insects and tend to anticipate complete eradication of pests in their dwellings, especially the medically and structurally important urban pests, the control of which contributes to a high degree of insecticide dependency [5]. For example, insecticide use in the U.S. accounted for 40% of total world use by volume in 2006, and at least 9% or 31.75 million kg (70 million lb) of these insecticides were applied in urban settings [4,6]. Unfortunately heavy insecticide use in urban environments causes increased selection pressure and thus has led to widespread development of insecticide resistance. Based on the arthropod pest resistance database established and maintained by Whalon *et al.*, six (30%) of the top 20 insecticide resistant arthropods are urban pests [7] (Table 1).

**Table 1:** Top 20 resistant arthropods in agricultural and urban ecosystems [7] (permitted kindly by Drs. Mark Whalon and David Mota-Sanchez).

Rank	Common Name	Scientific Name	Number *	Ecosystem
1	Two-spotted spider mite	*Tetranychus urticae*	94	Agricultural
2	Diamondback moth	*Plutella xylostella*	92	Agricultural
3	Green peach aphid	*Myzus persicae*	76	Agricultural
4	House fly	*Musca domestica*	62	Urban
5	Colorado potato beetle	*Leptinotarsa decemlineata*	55	Agricultural
5	Sweetpotato whitefly	*Bemisia tabaci*	55	Agricultural
7	Southern cattle tick	*Rhipicephalus microplus*	50	Agricultural
8	Cotton aphid	*Aphis gossypii*	49	Agricultural
9	Corn bollworm	*Helicoverpa armigera*	48	Agricultural
9	European red mite	*Panonychus ulmi*	48	Agricultural
11	German cockroach	*Blattella germanica*	42	Urban
12	Southern house mosquito	*Culex quinquefasciatus*	40	Urban
13	Beet armyworm	*Spodoptera exigua*	38	Agricultural
13	Oriental leafworm moth	*Spodoptera litura*	38	Agricultural
15	House mosquito	*Culex pipiens pipiens*	36	Urban
16	Yellow fever mosquito	*Aedes aegypti*	35	Urban
16	Tobacco budworm	*Heliothis virescens*	35	Agricultural
18	Hop aphid	*Phorodon humuli*	34	Agricultural
19	Red flour beetle	*Tribolium castaneum*	33	Urban
20	African cotton leafworm	*Spodopotera littoralis*	30	Agricultural

* Number of active ingredients to which the pest has exhibited documented resistance.

The development of insecticide resistance is a dynamic and complex process, depending directly on genetic, physiological, behavioral and ecological factors of the arthropod pests, and depending indirectly on operational factors including categories of insecticides used as well as the application timing, rate, coverage and method [8,9]. Insecticide resistance management (IRM) strategies in urban ecosystems consist of understanding the status and mechanisms of insecticide resistance, overcoming or delaying resistance to existing compounds and preventing the development of resistance to new pesticides through reducing the insecticide selection pressure [8,10]. In this review we will focus on the most commonly used insecticides and their molecular and physiological mechanisms in six major urban insect pests: house fly, German cockroach, mosquitoes, red flour beetle, bed bugs and head louse. We will also discuss several promising approaches that prove suitable for inclusion in future urban IPM programs.

## 2. Insecticide Resistance in Six Major Urban Insect Pests

Insecticide resistance is a fundamental threat to global urban pest management [11]. In order to design more sustainable IRM strategies, it is essential to identify the insecticides applied for urban pest control and to gain a complete understanding of the phenotypic and genotypic mechanisms underlying resistance developed by the pests.

### 2.1. House Fly

The house fly (*Musca domestica* L.), is a cosmopolitan urban pest long associated with humans and domesticated animals. House flies are very well adapted to a wide variety of human habitations including housing, garbage dumps, animal shelters and food storage and delivery facilities. House flies persist from tropical to temperate climates in developing and developed countries [12]. While the house fly is often considered an annoyance pest, it is also a notorious vector that can transmit more than 100 human and animal diseases caused by many deadly antibiotic-resistant zoonotic pathogens [12,13]. The mobility and feeding characteristics of the house fly as well as its role in transmitting diseases make it an increasing public health threat in the urban environment.

House fly management typically requires multiple applications of insecticides. Unfortunately, the house fly has a well-documented history of developing resistance to many insecticides, including pyrethroids, neonicotinoids, organophosphates (OPs), carbamates, organochlorines and the triazine cyromazine [14,15,16,17]. The house fly has been found to be resistant to 62 unique insecticide active ingredients, with 337 documented cases worldwide, and is listed as the world’s No. 1 resistant urban insect pest [7] (Table 1).

In house fly control, pyrethroids remain the most extensively used synthetic insecticide class due to several factors including product efficiency, vertebrate safety, extended residual activity and relatively low cost. As a result, house flies have developed resistance to pyrethroids all over the world [15,16,17,18]. Two major pyrethroid resistance mechanisms have been documented in house flies: cytochrome P450 monooxygenase (P450)-mediated detoxification [19,20,21,22] and target site insensitivity [16,23,24,25]. The elevated expression of multiple P450s has been shown to connect with pyrethroid resistance in house flies, including *CYP6D1* in the LPR strain [26,27], *CYP6A1* in the Rutgers strain [28], and *CYP4D4v2*, *CYP4G2*, *CYP6A5v2*, *CYP6A36*, *CYP6A38* in the ALHF strain [29,30,31]. Three mutations, *kdr* (L1014F), *kdr-his* (L1014H) and *super-kdr* (M918T + L1014F) have been identified in the voltage-gated sodium channel (*VGSC*) of pyrethroid-resistant house flies [16,32]. The function of these mutations in pyrethroid resistance has been confirmed by electrophysiological studies [16,23,33]. These mutations have also been evaluated in many field populations globally with frequencies varying among locations [15,16,24].

OP and carbamate insecticides were commonly applied for house fly control from the 1960s to 1990s in the USA [34]. Nowadays, these chemicals are continuously being used in many areas of the world, especially in developing countries [15]. Resistance of house flies to OPs or carbamates has been linked to mutations in the acetylcholinesterase gene (*Ace*) [34,35] or associated with reduction in the activity of a carboxyesterase (*MdαE7*) [36]. There were six mutations in *Ace* identified from resistant house fly strains [34], four of which (V260L, G342A/V, F407Y), along with two *MdαE7* mutations (W251L/S), were commonly present in field house fly populations collected from China [15].

Imidacloprid, a neonicotinoid targeting nicotinic acetylcholine receptors (nAChRs), is highly effective against many insect pests including the house fly [37]. Genetic studies suggested imidacloprid resistance found in a Pakistani house fly population was autosomal, incompletely recessive and polygenic [38]. In a study with Danish house fly strains, the up-regulation of two P450s, *CYP4G2* and *CYP6G4,* was suggested to play a role in the resistance to neonicotinoids [17].

House flies have also developed resistance to many other insecticides, as well as biopesticides or biorational insecticides and insect growth regulators used for control. Examples include the insecticides spinosad [39,40,41], indoxacarb [42] and cyromazine [43,44,45].

### 2.2. German Cockroach

The German cockroach, *Blattella germanica* (L.), is a common indoor cockroach species. Infestations of this pest are associated with poor sanitation, particularly in and around food-handling facilities, and also tend to be associated with lower socioeconomic status. Cockroach infestations lead to food damage and contamination because cockroaches can vector human and domesticated animal pathogens. Cockroach feces, saliva and cast skins contain allergens that may trigger allergic reactions and psychological distress in sensitive individuals [46]. Cockroaches are among the most problematic urban pests in initiating asthmatic and allergic reactions in children [47].

Cockroach management relies extensively on insecticide application; insecticide baits are the most popular and efficient formulation [48]. The German cockroach has been reported to have developed resistance to 42 unique insecticide active ingredients in 219 documented cases worldwide and is ranked as the world’s No. 2 insecticide resistant urban pest [7] (Table 1). Conventional cockroach control programs have used spray formulations containing carbamates, OPs, organochlorines and pyrethroids. Consequently, high levels of resistance to these insecticides have been documented in many field populations [49,50,51,52,53,54,55].

The mechanisms of insecticide resistance in German cockroaches include behavioral resistance, target site insensitivity and metabolic detoxification [56]. The resistance mechanism can be different in different populations. For example, synergist studies have demonstrated that the resistance to pyrethrins (9.5-fold) in a field-collected Kenly strain was not affected by the synergists piperonyl butoxide (PBO, inhibitor for P450s) or S,S,S-tributylphosphorotrithioate (DEF, inhibitor for esterases), indicating that neither P450 nor esterase-mediated metabolic detoxification were involved in the reported resistance [49]. Conversely, the malathion resistance in a field Rutgers strain was suppressed with PBO, suggesting that P450-mediated detoxification was involved [49]. In another example, the pyrethroid resistance in the Ectiban-R strain was closely associated with a single mutation in the *VGSC*, but not with P450 or esterase activity [49,57]. Although three German cockroach populations, Apyr-R, Bpyr-R and Cpyr-R shared the same geographic origin, they also exhibited diverse pyrethroid resistance mechanisms [58]. It was reported that P450-mediated metabolic detoxification played minor roles in Bpyr-R and Cpyr-R, while it played a very important role in Apyr-R [58,59].

Since the mid-1980s, insecticide baits have been used to successfully control infestations of German cockroaches, largely replacing broadcast liquid spray treatments [48]. Baits fit well into urban IPM programs, both increasing control efficacy and significantly reducing impacts on non-target organisms, making them appropriate for use in insecticide-sensitive environments [60]. Moreover, baits have been shown to reduce cockroach allergens to below clinically significant levels [61]. In a study comparing conventional and bait-based IPM programs in a school situation, the precise placement of insecticide baits in infested areas decreased insecticide use by 275% and nearly eliminated student exposure to the insecticides [48]. The insecticides used in bait formulations include fipronil, indoxacarb, imidacloprid, dinotefuran, abamectin, hydramethylnon and abamectin + pyriproxyfen [48,62]. Generally baits would be considered more effective than sprays, because they deliver a higher insecticide dose, which may prevent the development of physiological resistance through reducing exposure to sublethal doses of insecticides [60]. However, proactive resistance monitoring with bait insecticides remains necessary. A recent study reported that a field-collected Gainesville-Resistant (GNV-R) strain displayed approximately 38-fold resistance to topically applied fipronil compared to the susceptible strain [60]. P450-mediated detoxification and target-site insensitivity mechanisms were suggested to confer resistance to fipronil in the GNV-R strain [63]. Reduced susceptibility to indoxacarb was also detected in more than half of 14 field German cockroach strains tested [64].

Besides physiological resistance to bait insecticides, German cockroaches have developed behavioral resistance to various phagostimulants of bait formulations, typically D-glucose and D-fructose [65,66,67]. The glucose aversion in field German cockroach populations resulted in the failure of attracting cockroaches to toxic baits and protected them from receiving lethal doses of insecticides [65,66].

### 2.3. Mosquitoes

As obligate blood feeders, many mosquito species harbor and transmit human disease pathogens [68]. For example, *Anopheles* mosquitoes contribute to the transmission of malaria parasites (*Plasmodium* spp.), which are among the top causes of death worldwide (198 million malaria infections resulted in 584,000 deaths in 2013) [68]. *Aedes* mosquitoes, such as *Ae. aegypti* and *Ae. albopictus*, can transfer dengue fever, yellow fever and chikungunya fever viruses [69]. The recent resurgence of dengue threatens 40% of the world’s population, with approximate 50–100 million cases every year [69]. Species in the genus *Culex* transmit West Nile virus, St. Louis encephalitis virus, Japanese encephalitis virus, and the avian malaria parasite, all of which dramatically affect public health [70,71].

Since the introduction of dichlorodiphenyltrichloroethane (DDT) in 1940s, vector control has played a very important role in reducing the global burden of mosquito-borne diseases [72]. Malaria control has improved dramatically during the past decade; deaths attributed to malaria have decreased by one third, due in large part to insecticide-based strategies including indoor residual spraying (IRS), insecticide-treated nets (ITNs) and long-lasting insecticide-treated bednets (LLITs) [72,73]. Currently, there are only four classes of insecticides with two modes of action recommended by the World Health Organization (WHO) for IRS: pyrethroids, organochlorines, carbamates and OPs [72,74]. Pyrethroids and DDT share the VGSC as a target site. Both carbamates and OPs inactivate the enzyme acetylcholinesterase in the nervous system [71,74,75]. Pyrethroids are the only class of insecticide approved by the WHO for use in ITNs and LLITs [72]. The limited choice of insecticides and increasing insecticide resistance put current global disease vector control at risk.

As an insecticide with both repellent and killing functions, pyrethroids are the mainstay of current mosquito management. The use of only a single class of insecticides, however, dramatically increases the potential for resistance development [73,76]. Pyrethroid resistance in *Anopheles* mosquitoes is widespread in many countries of Africa [77,78]. Pyrethroid resistance in *Culex* mosquitoes has also been identified worldwide [79]. *Aedes* mosquitoes are reported to show pyrethroid resistance in populations from Singapore, Thailand, Malaysia, Brazil, Mexico and Colombia [80].

So far four mechanisms have been reported in pyrethroid resistance of mosquitoes [56,72,76,77]. *Kdr*-mediated target site insensitivity is one of the most common mechanisms. In total, 11 VGSC mutations have been identified in mosquitoes, six of which have been functionally examined in *Xenopus* oocytes [33,71]. Several mutation combinations were also linked to pyrethroid resistance in mosquito populations [71,81]. The second mechanism is enhanced metabolic detoxification [76,82,83,84,85]. For example, increased expression of a number of P450s had been confirmed to contribute to pyrethroid resistance or cross-resistance between pyrethroids and other insecticides. A P450, *CYP6P3*, detected from *An. gambiae* was significantly overexpressed in field-caught permethrin-resistant mosquitoes, and the CYP6P3 protein could metabolize both permethrin and deltamethrin [86]. CYP6M2 can metabolize both pyrethroids and DDT, causing cross-resistance between these two classes of insecticides in wild *An. gambiae* mosquitoes in Ghana [87]. Similarly, up-regulation of two P450s, *CYP6Pa* and *CYP6Pb*, was suggested to be the primary mechanism responsible for pyrethroid resistance in field *An. funestus* populations of southern Africa [88]. Two recent studies demonstrated that multiple P450s or multiple gene families (including P450s, esterases, cell transporters and cuticular components) may contribute to pyrethroid resistance in a single population [89,90]. The third major resistance mechanism is decreased cuticular penetration [77]. As the first line of defense against insecticides, a thicker cuticle leads to a slower rate of insecticide absorption and penetration, which reduces the uptake of insecticides. In an *An. funestus* population collected from southern Mozambique, pyrethroid resistance was associated with an increased cuticle thickness [91]. The temporal and spatial expression of three cuticular proteins in *An. gambiae* revealed the potential function of two proteins (CPLCG3 and CPLCG4) in slowing penetration of insecticides and a third protein (CPF3) in increasing the desiccation tolerance [92]. Recently, a functional genomics study revealed that cuticular proteins were associated with deltamethrin resistance in laboratory and field populations of *C. pipiens pallens* [93]. Furthermore, evidence suggests that behavioral resistance also plays a role in reducing the efficacy of insecticide treatment [77]. Genetic changes in mosquito populations may result in decreasing the chance of contacting insecticides through modified feeding and resting activities [94,95,96].

Besides pyrethroid insecticides, OP, carbamate and organochlorines (e.g., DDT) are commonly used in IRS against pyrethroid-resistant vectors. Data collected from 125 countries during 2000 to 2009 showed that DDT remained the primary insecticide used for vector control in terms of quantity (71% of all pesticides used, by volume), geographically concentrated in India and Africa [97]. Although DDT shares the same mode of action with pyrethroids, some mosquito species have developed resistance to pyrethroids but remain susceptible to DDT [98,99]. Recent studies suggested that a single mutation in the upregulated glutathione S-transferase (GST) gene *GSTe2* was responsible for the high level of DDT and permethrin resistance in *An. funestus* mosquitoes of West and Central Africa [100]. In East Africa, metabolic detoxification played a major role in the DDT resistance of *An. funestus* mosquitoes [101]. In the malaria vector *An. gambiae* from Benin, DDT resistance was correlated with high frequency of sodium channel mutations and overexpression of two metabolic detoxification genes (*GSTe2* and *CYP6M2*) [102,103]. In addition, the high GST expression level in *An. culicifacies*, *An. annularis* and *Ae. aegypti* was also linked to DDT resistance in mosquitoes collected from India and Singapore [80,104]. To combat/delay insecticide resistance, carbamates and OPs have become increasingly important for IRS in combination or rotation with pyrethroids and DDT. However, some *An. gambiae* populations in Tiassalé and West Africa have already shown resistance to all of these insecticides [105,106]. Resistance to the most commonly used carbamate, bendiocarb, in *An. gambiae* mosquitoes in Tiassalé was associated with elevated expression of P450s, and duplication of the acetylcholinesterase gene *Ace-1*, as well as additional copies of the resistant *Ace-1* G119S alleles, which is a newly identified mechanism conferring bendiocarb resistance to *An. gambiae* [106]. OP resistance in mosquitoes was mainly associated with elevated levels of esterases [107,108] or *Ace-1* mediated target site insensitivity [106,109].

There are several relatively new insecticides/biopesticides with different modes of action used for mosquito larva management, such as fipronil, spinosad, imidacloprid, novaluron, methoprene and *Bacillus thuringiensis* [74,110]. They could serve as good alternatives for mosquito control, particularly when they are directed towards the aquatic larval stages.

### 2.4. Red Flour Beetle

The red flour beetle, *Tribolium castaneum* (Herbst), is a worldwide pest of stored grains, causing postharvest losses of up to 9% in developed countries and >20% in developing countries [111]. As an external-feeding pest or secondary pest, the red flour beetle attacks damaged grains or farinaceous materials in both larval and adult stages and readily adapts to stored-grain environments due to its high fecundity rates and relative longevity [112]. *T. castaneum* has a notorious and well-documented reputation for developing resistance to all classes of insecticides and fumigants used to control it [113]. *T. castaneum* ranks 19^th^ among the top 20 most insecticide-resistant arthropods with 132 recorded cases of insecticide resistance reported [7] (Table 1). A field-derived strain of *T. castaneum* (QTC279) exhibited a high level of deltamethrin resistance in one study. This resistance was almost completely suppressed by a P450 inhibitor, PBO, suggesting that P450-mediated detoxification was the major mechanism involved in the deltamethrin resistance [114]. Recently, a microarray study comparing the QTC279 strain with a susceptible Lab-S strain revealed six up-regulated P450 genes in the QTC279 strain [115]. Further functional genomics and reverse genetic approaches were used to determine that one P450, *CYP6BQ9*, was responsible for the majority of deltamethrin resistance in the QTC279 strain [115]. The other three P450s in the same cluster as *CYP6BQ9* (*CYP6BQ8*, *CYP6BQ10*, and *CYP6BQ11*) may also play minor roles in deltamethrin resistance [115,116]. Most recently, Liang *et al.*, established the transcriptional expression profiles of eight P450s in response to four different insecticides in a susceptible Georgia-1 strain, illustrating their potential roles in xenobiotic metabolism [117].

Phosphine gas (hydrogen phosphine) is the most commonly used fumigant for red flour beetle control worldwide. Phosphine gas has many positive attributes: it is relatively inexpensive to produce, easy to apply and leaves minimum residues [118,119]. However, resistance to phosphine has been reported in *T. castaneum* field populations from many countries due to the heavy selection pressure following consistent, year-after-year fumigations [119,120,121,122,123]. Commercial storage facilities in Oklahoma, USA have been reported to receive fumigation treatments on an average of 2.6 times a year [119]. So far, two phosphine-resistant phenotypes have been identified in *T. castaneum* populations, weak resistance and strong resistance. Next-generation sequencing and genetic studies revealed that two unknown gene loci (tc_*rph1* and tc_*rph2*) were associated with high levels of phosphine resistance [124]. Recent evidence suggests that several polymorphisms of a metabolic enzyme, dihydrolipoamide dehydrogenase (DLD) contribute to the phosphine resistance in *T. castaneum* and *Caenorhabditis elegans* [125]. These polymorphisms of DLD permit the development of a simple and robust molecular diagnostic method to monitor phosphine resistance in field *T. castaneum* populations [126].

The development and adoption of biologically derived insecticides such as spinosad, neem, pyrethrum and methoprene, as a next generation “green” treatment, provide opportunities to counter the problem of existing resistance [111,127].

### 2.5. Bed Bugs

Bed bugs have a long association with humans. Bed bugs are thought to have host-switched from bats to humans at least 10,000 years ago when early humans shared caves with bats [128]. The common bed bug, *Cimex lectularius* L., is a nocturnal, bloodsucking ectoparasite in the family Cimicidae within the order Hemiptera. Among approximately 90 species in the family Cimicidae, only two species, *C. lectularius* and *C. hemipterus* (tropical bed bug), rely on humans as primary host. *C. lectularius* is the most common bed bug species in the U.S., Australia, Europe, Asia and Africa [129,130]. *C. hemipterus* occurs mainly around 30° north and south latitudes and has been reported in Asia, Australia and Africa [130,131,132]. Bed bugs present a significant human health hazard. Bed bug bites often cause delayed skin irritations and sometimes lesions owing to host immune allergic responses, which potentially lead to infection [133]. Although there is no evidence showing bed bugs can transmit human disease, they are capable of harboring many human pathogens and viruses, including the filarial nematodes *Wuchereria* and *Brugia*, hepatitis B, C and E viruses, human immunodeficiency virus (HIV), *Coxiella burnetii* (the agent of Q fever), methacillin-resistant *Staphylococcus aureus* (MRSA), vancomycin-resistant *Enterococcus faecium* (VRE) and *Burkholderia multivorans* (a pathogen in nosocomial infections of patients with cystic fibrosis) [130,134,135]. Bed bug infestations can also cause human mental health problems, such as posttraumatic stress disorder (PTSD) stemming from associated insomnia, emotional distress, anxiety, stress, anger, embarrassment, paranoia and depression [130,136]. In addition, bed bug infestation results in social stigma and can cause economic hardship due to the cost of extermination and occasional need to replace infested furniture (the latter not as common with the advent of heat and other non-chemical control techniques, but still a factor in some situations) [137].

Archeologists have detected bed bugs in Egyptian tombs dating back more than 3500 years [138]. In the early 1900s, bed bugs became a year-round problem when central heating of buildings started to develop in Western societies [139,140,141]. The development and wide-scale use of DDT and other long-residual organochlorine insecticides in the 1940s provided the first effective control of bed bug populations. This widespread use of organochlorine insecticides resulted in bed bugs becoming uncommon in developed countries. Presently, DDT is still used extensively in many tropical countries and consequently resistance has developed to DDT in these regions [141]. In recent years, bed bug outbreaks have been reported from every state and territory in the U.S. [130]. Besides the U.S. and the U.K. [137,142,143,144], bed bugs have also rapidly resurged in other European countries [145], the Middle East [146], Australia [147], South America [148] and Asia [145,149,150]. Multiple factors are contributing to this sudden resurgence, including the ubiquitous development of resistance to commercially available synthetic insecticides that are permitted within structures where fear of human exposure is substantial [141,144,151,152,153].

Recent research is providing solid evidence that bed bug populations have developed resistance to commonly used pyrethroids. Two mutations, V419L and L925I, in the VGSC α-subunit gene have been identified as responsible for deltamethrin resistance in bed bugs [154,155]. Subsequently, these mutations (either one or both) were detected in 88% of populations collected from the Eastern U.S. [156], 100% of populations tested in a suburb of Paris [157], 96% of populations collected from Australia [158], and 100% of populations from Israel [159], suggesting that *kdr*-mediated pyrethroid resistance in *C. lectularius* is widespread throughout the world. A recent study has identified a new sodium channel mutation, I936F, in some populations of bed bugs collected in Australia but not in samples collected in Israel [158,159]. However, the function of this mutation remains unknown [158]. Additionally, four novel sodium channel mutations were detected in *C. hemipterus* populations collected from multiple countries [132]. Besides *kdr*-mediated target site insensitivity, other mechanisms have also been reported in pyrethroid resistance of bed bugs. For example, the CIN-1 population showed >2588-fold resistance to deltamethrin and no mutations were identified in the *VGSC* gene [156]. However, the deltamethrin resistance of this population was significantly suppressed by a P450 inhibitor, PBO, suggesting that P450-associated metabolic detoxification may play a role in the pyrethroid resistance of CIN-1 [160]. In a subsequent study, suppression of the expression of NADPH-P450 reductase (a partner enzyme of all P450s) increased deltamethrin susceptibility in pyrethroid-resistant populations, further confirming that P450-mediated detoxification was one of the resistance mechanisms [161]. Recently, next-generation sequencing studies have facilitated genome-wide analyses of insecticide resistance-associated genes in bed bugs and suggested that multiple mechanisms may contribute to pyrethroid resistance simultaneously in a single population [162,163,164]. These mechanisms include increased metabolic detoxification through up-regulation of P450s, esterases, GSTs and ATP-binding cassette (ABC) transporters, along with decreased cuticular penetration [165]. More recently, a functional genomics study provided evidence for the involvement of multiple mechanisms in pyrethroid resistance among field bed bug populations. In over 70% of the field-collected bed bug populations tested, all of the previously described mechanisms of resistance were detected [166]. Remarkably, most of these resistance-associated genes were expressed in the cuticle, where the insecticides can be detoxified or blocked before reaching the target site (sodium channel) in nerve cells [166].

In the light of ubiquitous pyrethroid resistance in bed bug populations, recent attention has been paid to non-pyrethroid insecticides for use against bed bugs. For example, dual-action insecticides combining pyrethroids with neonicotinoids [167] are showing varying effectiveness on field-collected populations [168]. In addition, both chlorfenapyr [169] and a juvenile hormone analog formulation (active ingredient (*S*)-hydroprene) [170] exhibited effectiveness on pyrethroid-resistant bed bugs. Horizontal transfer of insecticidal dust and several botanical insecticides demonstrated that bed bug mortality could be caused by acquisition of insecticides from other exposed bed bugs [171]. Most recently, the effect of a fumigant (sulfuryl fluoride) was investigated [172]. At 15 °C, a target dose of 285 g-h/m^3^ resulted in 100% mortality of adults, late-instar nymphs and eggs [172]. Overall, these non-pyrethroid insecticides present promising options for controlling pyrethroid-resistant bed bugs.

### 2.6. Head Louse

The human head louse *Pediculus humanus capitis* (De Geer) (Anoplura: Pediculidae) is one of the most prevalent obligate parasites infesting humans worldwide [173]. Children are at increased risk and it has been reported that 8% of school-aged children in the U.S. have been infested. Eggs and juveniles of the head louse are known as “nits.” Many schools have a “no-nit” policy and children with infestations have to be removed from school [173,174]. The costs associated with head louse management are estimated to be approximately $1 billion annually in the U.S. alone. Additionally, this dollar amount does not quantify any social, mental, or economic impacts caused by missing school or by inefficient treatment [173,174,175].

Head louse control is largely based on physical removal (shaving or combing the hair) combined with the application of pediculicides. There are six major groups of pediculicides commercially used for control of head louse infestation through topical application. They are natural pyrethrin esters (pyrethrum), synthetic pyrethroids (permethrin, phenothrin), an organochlorine (lindane), an OP (malathion) and a carbamate (carbaryl) [173,176,177]. Among them, pyrethrins and synthetic pyrethroids remain the most common over-the-counter pediculicides since they became available in 1992. The first documentation of pyrethroid resistance was reported in France in 1994 followed by cases recorded from many other countries in Europe, North America, South America, Asia and Australia [173,177]. Pyrethrins and pyrethroids share a common target site (*VGSC*) on the neuron membrane with DDT. Three sodium channel mutations (M815I, T917I, and L920F) were identified on the *VGSC* gene associated with permethrin resistance in head lice [173,178]. The mutation T917I corresponded to the house fly mutation T929I, and its function had been characterized in *Xenopus laevis* oocytes [179]. The mutation T917I alone or combined with one or both of M815I and L920F led to a loss of permethrin sensitivity of VGSC, suggesting a vital role of the mutation T917I in permethrin resistance of head lice [179]. Singly mutated M815I and L920F (corresponding to house fly mutations M827I and L932F) variants also reduced the permethrin sensitivity of *VGSC* [179]. The allele frequencies of these mutations were examined in human head louse populations collected from 14 countries [180]. The results demonstrated that *kdr*-mediated pyrethroid resistance was wide-spread in head louse populations globally but the intensity varied substantially among countries [173,180]. A recent study investigated the extent and frequency of the principal mutation T917I in 32 populations collected from Canada and the U.S. [175]. The frequency of T917I increased dramatically in U.S. head louse populations from 84.4% to 99.6% over the course of a decade, while exhibiting a uniformly high pattern in Canadian populations (97.1% in 2008) [175]. Besides target site insensitivity, P450-mediated detoxification may also play a role in the pyrethroid resistance of head lice in some Israeli and Argentine populations [181,182].

Malathion is an OP insecticide targeting and inhibiting AChE which causes spastic paralysis and eventual death of insects. Malathion is not a commonly used pediculicide in the U.S. due to the prolonged application period required, flammability and environmental concerns. However, malathion has been consistently used in Europe for head lice control [176]. Malathion resistance was first reported in France in 1995 [183], in the U.K. in 1999 [184], in Australia in 2003 [185], and in Denmark in 2006 [186]. Low levels of malathion resistance were also reported in Florida and southern California in the U.S. [187]. In many insects, esterase-mediated detoxification is the major mechanism conferring resistance to malathion [188]. A carboxylesterase *HLCbE3* exhibited 5.4-fold higher transcriptional expression in a malathion-resistant BR-HL strain than in susceptible head lice [189]. Knockdown of *HLCbE3* expression through RNA interference (RNAi) in BR-HL head lice resulted in an increase of malathion susceptibility, indicating this carboxylesterase was responsible for the malathion resistance in the BR-HL head lice [189].

Due to the limited number of available pediculicides and widespread pyrethroid and malathion resistance, several new topical pediculicides have recently been introduced to the market for head lice control, including dimeticone, ivermectin, benzyl alchohol, and spinosad [190,191,192,193]. These pediculicides are of interest owing to their novel modes of action, low mammalian toxicity and little cross-resistance with commonly used groups of pediculicides [194]. To develop a proactive resistance monitoring approach, a non-invasive induction assay was optimized for identifying detoxification genes involved in resistance to ivermectin. Expression of three P450s (*CYP6CJ1*, *CYP9AG1*, *CYP9AG2*) and one ABC transporter gene (*PhABCC4*) were induced by ivermectin [195]. Knockdown of *CYP9AG2* or *PhABCC4* through RNAi led to increased sensitivity of head lice to ivermectin, suggesting these two genes were involved in ivermectin resistance [173,194,195].

## 3. Integrated Approaches Suitable for Urban Pest Management

Recent advances in genomic and genetic technologies have facilitated the development of alternative urban pest management strategies including investigation of biomarker-based insecticide resistance monitoring, genetic modification of wild pest populations and RNAi-based insecticides. Moreover, recent increasing development of biopesticides offers great potential to reduce the use of synthetic insecticides and enhance control efficiency in urban ecosystems. The combination of these alternative technologies with chemical control approaches that have been validated for effectiveness in urban pest management will optimize current urban pest control activities and may potentially delay the development of insecticide resistance.

### 3.1. Molecular and Biotechnological Approaches

#### 3.1.1. Molecular Markers

Proactive insecticide resistance monitoring is an integral part of IPM programs. Knowledge of pest susceptibility to insecticides, observing and tracking resistance trends and understanding mechanisms of resistance are the basis for building the insecticide application component of a pest control program. A resistance management strategy with molecular markers is crucial because it can monitor insecticide resistance before it reaches the tipping point and the effectiveness of insecticides is diminished [72,173]. Compared with traditional bioassay methods, a molecular method requires fewer insect samples and allows accurate and direct analysis of resistance-associated genes. For example, when the resistance allele is recessive and heterozygotes are abundant in the population, or the frequency of resistance is low, efficient detection of resistance by bioassays alone is often unachievable [72]. The molecular tests can be performed by polymerase chain reaction or sequencing techniques with DNA, or transcriptomic analysis with RNA. There are many molecular markers developed for resistance monitoring as we described in the previous section, including mutations on target genes such as VGSC, AChE, DLD [32,106,126,156,180]; and up- and down-regulation of various resistance-associated genes [89,166].

#### 3.1.2. Genetically Modified Insects

The core of genetic modification of insect pests is through introduction of a heritable element into a target population to enhance pest control. The successful application of this approach would provide ecologically benign, species-specific population management for target insect pests [196]. The classic genetic modification is the Sterile Insect Technique (SIT) initiated by U.S. entomologist Edward F. Knipling in 1955 [197] and improved by the International Atomic Energy Agency and the Food and Agricultural Organization of the United Nations [198]. The classic SIT sterilizes males by the application of irradiation [199]. Upon the release of a large number of sterilized males, SIT results in few fertile males successfully competing for mates. When sterilized males mate with wild females, infertile eggs result and the wild populations are eventually reduced to low levels or completely eliminated. Up to now, SIT has been applied for suppression or local eradication of several key agricultural and urban insect pests, including the screwworm fly (*Cochliomyia hominivorax*) in North America and the tsetse fly (*Glossina* *fuscipes*) in Zanzibar [198,199,200,201,202]. However, insects’ fitness can be reduced after exposure to the damaging doses of radiation, causing irradiated individuals to show reduced mating success compared to wild males [196].

With modern molecular and genetic technologies, two strategies are being used to improve SIT approaches. One is release of insects carrying a dominant lethal gene (RIDL), and the other is homing endonuclease genes (HEGs) [203]. In RIDL, a construct with a female-specific promoter driving a lethal gene (e.g., flightless gene) results in a female-killing event in the F_1_ generation. Another RIDL construct is a stage-specific promoter driving a late-acting lethal gene leading to pupal or adult mortality in both male and female F_1_ offspring [196,203]. An open field release of RIDL mosquitoes was tested in the Cayman Islands recently and showed that engineered sterile male mosquitoes could mate with wild females and fertilize their eggs, suggesting the practicability of this technique in suppressing mosquito populations [204,205,206]. HEGs are selfish genetic elements discovered in bacteria and subsequently introduced into mosquitoes. HEGs encoding endonucleases recognize and insert themselves in the middle of specific genomic recognition sequences to protect themselves from self-degradation [203]. They also can be subsequently passed on through any offspring [203]. HEGs have been designed to knock out specific mosquito genes to generate pathogen-resistant females, induce sterility, and reduce fecundity or lifespan, or to disorder the population’s sex ratio, leading to suppression of the mosquitoes’ disease transmission capabilities or reduction in population abundance [207,208,209]. Instead of randomly introducing genetic modifications by DNA-damaging agents in classic SIT, the RIDL and HEG methods are more accurate and efficient. Engineered insects are also better able to compete for mates than those in the classic SIT scenario [196,203].

Currently, new genome-editing technologies, including zinc-finger nucleases (ZFNs), transcription activator-like effector (TALE) nucleases (TALENs), and clustered regularly interspaced short palindromic repeats (CRISPRs), have become available [210]. They permit more specific and efficient genetic modifications. Development of these strategies could provide IPM practitioners with new methods of pest control [210,211,212]. ZFNs consist of double DNA-binding modules derived from natural transcription factors bound to the endonucleolytic domains of a Type IIS restriction enzyme, *Fok*I. TALENs utilize double DNA-binding modules from bacteria TALEs binding to the same *Fok*I cleavage domain [210]. A recent study reported that ZFN-mediated knock-out of an obligate odorant co-receptor (*Orco*) resulted in the reduction of odor response in *Ae. aegypti* [213]. ZFNs have also been used to mutate the *AaegGr3* gene encoding a subunit of the heteromeric CO_2_ receptor from *Ae. aegypti* mosquitoes [214]. This loss-of-function study revealed the role of *AaegGr3* in CO_2_ detection during host searching [214]. TALEN-mediate cleavage technology was used to disrupt an immunity gene *thioester-containing protein 1* (*TEP1*) from *An. gambiae*, leading to mutant mosquitoes that became hypersusceptible to infection by *Plasmodium* parasites, which opened a new avenue for malaria control [215]. The more recently developed CRISPR/Cas9-mediated gene modification system is composed of a single guide RNA (sgRNA), target DNA sequences and a multifunctional Cas9 protein for cleavage [210,211]. Compared with ZFN and TALEN tools, the CRISPR/Cas9 approach has many advantages in terms of ease and specificity of genetic modification. These advantages include only a single exogenous protein (Cas9) involved, easily designed and highly specific sgRNA and the ability to engineer multiple loci simultaneously by co-injection of multiple sgRNAs [210,211,216,217,218]. Most recently, CRISPR/Cas9 was used to knockdown a male-determining factor gene, *Nix*, in *Ae. aegypti* mosquitoes, leading to partial sex-change phenotypes [219]. This study provides a potential new mosquito control strategy by converting blood-feeding female mosquitoes into harmless males.

Although the current research into genome-editing technologies described here is mainly focused on mosquitoes, the tactics can likely be adapted to other urban insect pests. Genetically modified insects hold great potential to be used as alternatives to chemical control in urban ecosystems. However, the significant obstacles to the successful application of these genetic technologies, such as off-target effects as well as a number of ecological, environmental and regulatory issues need to be seriously considered [211,219].

#### 3.1.3. RNAi-based Insecticides

RNAi as a method of sequence-specific gene silencing has opened a new era for reverse functional genomics and genetic research in many eukaryotic organisms. The double-stranded RNA (dsRNA) mediated loss-of-function approach can be used as a new insecticidal tactic in combination with other existing tactics to manage insecticide resistance in both agricultural and urban ecosystems [220].

Development of RNAi insecticides could prove cost effective and environmentally benign due to its high specificity. RNAi has been experimentally deployed to target several urban and disease-vectoring insect pests including ants, bed bugs, cockroaches, head lice, mosquitoes, red flour beetles, sandflies, termites and tsetse flies [195,212,220,221,222,223,224,225]. In termites, sixteen genes were successfully silenced from host insects or their symbiotic protozoa, revealing potential targets for termite control [225]. The efficiency and convenience of delivery of dsRNA largely depend on the method of introduction of dsRNA into the insect cells or the insect body. Oral delivery of dsRNA allows automatic and constant uptake of dsRNA. Therefore, this method can be used to control pests in the urban environment. In controlled feeding RNAi studies, the dsRNA was either *in vitro* synthesized through enzymatic reverse transcription [226] or *in vivo* synthesized by a special *Escherichia coli* strain [227]. The latter approach holds potential for managing urban pests because of its cost effectiveness. For example, *E. coli* expressed dsRNAs of testis genes and a female sex determination gene were fed to mosquito larvae which reduced male fertility and helped produce a highly male-biased population of mosquitoes to enhance SIT tactics [227]. Moreover, a carrier system for delivery of dsRNA could significantly enhance the stability of dsRNA and increase the cellular uptake. Recently, a chitosan/dsRNA-based nanoparticle has been successfully delivered in *An. gambiae* mosquitoes [228]. The larvae feeding on RNAi showed that knockdown of two chitin synthase genes resulted in increased susceptibility to insecticides [228]. More recently, three nanoparticles, chitosan, carbon quantum dot and silica, complexed with dsRNA were evaluated in *Ae. Aegypti*, and it was found that chitosan and carbon quantum dot were efficient delivery methods [229].

### 3.2. Biopesticides

Biopesticides derived from living microorganisms or natural products, marketed as “green chemicals”, have earned some market share as an alternative tactic for pest control and for insecticide resistance management of urban pests. Biopesticides have been estimated to have a five-year annual growth rate of 16%, and are projected to encompass a $10 billion global market by 2017 [230]. Compared with synthetic insecticides, biopesticides often have several advantages that make them suitable for pest control in urban ecosystems: (1) Biopesticides are often effective and usually have specificity against their target insects with limited impacts on non-target organisms [231,232]; (2) Biopesticides typically are biodegradable and have low risk of accumulating in the environment [232,233,234]; (3) The active ingredients of biopesticides typically have biologically variable structures and modes of action, which help inhibit the development of insecticide resistance [233].

The U.S. Environmental Protection Agency (EPA) classifies biopesticides in three categories. They are microbial biopesticides, biochemicals and semiochemicals [230]. Microbial biopesticides include bacteria, fungi, oomycetes, viruses and protozoa. An infection of entomopathogenic fungi from the Hyphomycetes (*Beauveria bassiana* and *Metarhizium anisopliae*) resulted in increased mosquito mortality after blood feeding and reduced the survivorship of malaria parasites inside the mosquito [235,236]. These microbial insecticides also function synergistically with various synthetic insecticides, suggesting the potential for the incorporation of fungal biopesticides in malaria control programs [237,238,239,240]. In another study, recombinant *M. anisopliae* strains expressing salivary gland and midgut peptide 1 were introduced into mosquitoes and successfully inhibited malaria parasite development [241]. This method could also serve as a tool to combat malaria. Endosymbiotic bacteria constitute the other type of microbial biopesticides. For example, *Wolbachia pipientis* is a well-studied endosymbiotic bacterium that is transmitted vertically from mother to offspring and is responsible for a number of reproductive disorders in their hosts including cytoplasmic incompatibility (CI) [242]. CI leads to unhatched eggs when a *Wolbachia*-infected male mosquito mates with an uninfected female. *Wolbachia*-infected females produce infected offspring when they mate with either uninfected or infected males, allowing the *Wolbachia* infection to spread rapidly through populations [203,243]. To date, *Wolbachia* had been successfully established in many malaria hosts such as *Ae. aegypti*, *Ae. albopictus* and *An. stephensi* by embryonic microinjection of *Wolbachia* purified from infected hosts [242,244,245,246]. Further studies on the field release of *Wolbachia*-infected mosquitoes are in progress currently [203]. In bed bugs, *Wolbachia* has been recognized as a bacteriocyte-associated nutritional mutualist [247]. Eliminating the *Wolbachia* endosymbiont from bed bugs to disturb their normal growth and reproduction is a potential biocontrol strategy.

Biochemical biopesticides comprise plant secondary metabolites that deter herbivorous insects from feeding on plants [230,248]. In recent years, essential oils derived from aromatic plants have gained increased attention as tools for pest control in urban IPM programs [233,234]. Amer and Mehlhorn reported on the repellency of 41 essential oils against *Aedes*, *Anopheles* and *Culex* mosquitoes [249]. A review of literature by Dhang and Sanjayan listed many essential oils and other plant products used for cockroach, house fly and termite control [234]. Moreover, essential oils are also promising candidates for inclusion in a bed bug IPM program. An essential oil-based biopesticide consisting of a blend of geraniol, cedar extract and sodium lauryl sulfate has demonstrated effectiveness at killing bed bugs. This product, marketed under the trade name EcoRaider, demonstrated the greatest efficacy among all biopesticides tested on bed bugs to date [250,251]. Bed bugs at all motile stages that were covered in a direct spray of EcoRaider exhibited 100% mortality. A recent field experiment reported that there was no significant difference in bed bug reduction between treatments with EcoRaider and with a synthetic insecticide, Temprid SC, indicating this essential oil-based biopesticide has potential to be used in bed bug IPM programs [252].

Semiochemicals are chemical signals produced by one organism and used for communication among individuals of the same species or different species. The most commonly used semiochemicals for urban pest control are insect pheromones. Most insect pheromones have been synthesized for monitoring of stored-product pests, trapping cockroaches and bed bugs, or mating disruption programs [253,254,255].

### 3.3. Combination of Multiple Approaches

Pest management in urban ecosystems will benefit from greater knowledge of the biology of target pests. Besides the tools described above, a number of other approaches based on biology, behavior and ecological factors have been developed for the control of several urban pests. For example, successful techniques to attract bed bugs and monitor bed bug infestations, including traps baited with carbon dioxide, heat and/or chemical lures have been developed [256,257,258]. Monitoring for pests improves the efficiency of pest control and eliminates unneeded prophylactic insecticide sprays. Physical treatments like replacement of wood bed frames with metal ones, mattress encasements and extreme temperature management are effective practices for current bed bug control [145]. All these non-chemical approaches could play significant roles in reducing insecticide selection pressure. However, no single approach is a panacea to solve the problem of insecticide resistance. A sustainable integrated IRM strategy requires the use of insecticides with multiple modes of action applied in space and time (rotations and mosaics) and the use of insecticide mixtures in concert with as many other approaches as possible. A recent review suggested that an integrated pest management strategy combining chemical and non-chemical approaches has been proved to be the best tactics for bed bug management based on the long-term research [259].

## 4. Conclusions

In urban ecosystems, developing sustainable IRM strategies relies on continued investigation of the status and mechanisms of insecticide resistance as well as understanding the biology, behavior, physiology and ecology of the target insect pests. Many operational factors such as categories of insecticides used, the application time, rate, coverage and method are also very important in designing IRM strategies. Recent advances in genomic and genetic technologies have facilitated the development of alternative tools that provide great potential for ecologically benign and species-specific insect population management. Moreover, biopesticides have varied mechanisms of activity that could contribute additional defenses against the development of insecticide resistance. There is no single technology that will provide a comprehensive solution for IRM. IRM must incorporate multiple tactics to achieve acceptable urban pest management and reduce the development of insecticide resistance.
